# Combining surgery and immunotherapy: turning an immunosuppressive effect into a therapeutic opportunity

**DOI:** 10.1186/s40425-018-0398-7

**Published:** 2018-09-03

**Authors:** Orneala Bakos, Christine Lawson, Samuel Rouleau, Lee-Hwa Tai

**Affiliations:** 1 Department of Anatomy and Cell Biology, Faculty of Medicine and Health Sciences, Université de Sherbrooke, Sherbrooke, QC Canada; 2 Centre de Recherche Clinique de Centre Hospitalier de l’Université de Sherbrooke (CHUS), Room 4853, 3001, 12e Avenue Nord, Sherbrooke, Québec J1H 5N4 Canada

**Keywords:** Perioperative period, Postoperative immunosuppression, Perioperative immunotherapy

## Abstract

**Background:**

Cancer surgery is necessary and life-saving. However, the majority of patients develop postoperative recurrence and metastasis, which are the main causes of cancer-related deaths. The postoperative stress response encompasses a broad set of physiological changes that have evolved to safeguard the host following major tissue trauma. These stress responses, however, intersect with cellular mediators and signaling pathways that contribute to cancer proliferation.

**Main:**

Previous descriptive and emerging mechanistic studies suggest that the surgery-induced prometastatic effect is linked to impairment of both innate and adaptive immunity. Existing studies that combine surgery and immunotherapies have revealed that this combination strategy is not straightforward and patients have experienced both therapeutic benefit and drawbacks. This review will specifically assess the immunological pathways that are disrupted by oncologic surgical stress and provide suggestions for rationally combining cancer surgery with immunotherapies to improve immune and treatment outcomes.

**Short conclusion:**

Given the prevalence of surgery as frontline therapy for solid cancers, the emerging data on postoperative immunosuppression and the rapid development of immunotherapy for oncologic treatment, we believe that future targeted studies of perioperative immunotherapy are warranted.

## Background

Solid cancers are the second leading cause of death worldwide, accounting for 8.8 million deaths in 2016. The most common causes of solid cancer death are cancers of lung (1.69 million deaths), liver (788,000 deaths), colorectal (774,000 deaths), stomach (754,000 deaths) and breast (571,000 deaths) (WHO statistics). Major thoracic or abdominal surgery is the mainstay of treatment for these top 5 solid cancers to extend the patient’s life. Unfortunately, disease recurs within 5 years in the majority of these patients and they tend to not respond to frontline therapies [1]. Minimal residual disease are occult tumors that persist in the patient following curative surgery.

Since the initial observation of the prometastatic effects of surgery by surgeons in 1913 [2], numerous preclinical tumor models have demonstrated that surgical resection contributes to the development of metastatic disease [3, 4] with the frequency of metastatic deposits correlating with the degree of surgical trauma [3]. Despite these early promising findings, limited mechanistic advances have been made. In clinical studies, complications in the postoperative period have been shown to associate with increased development of metastatic disease and poor cancer survival [5, 6]. Various perioperative changes have been proposed to describe the promotion of metastases following surgery, including tumor cell dissemination into nearby blood vessels and lymphatics [7, 8], local and systematic release of growth factors [9, 10], and cellular immune suppression [11–15].

There is increasing mechanistic evidence to suggest primary tumor surgical resection disrupts the host immune system. These effects lie within the “postoperative period”, which lasts days [16] to weeks [16, 17] following tumor surgical resection and has been suggested to create an immunosuppressive window for the expansion and escape of occult tumors [11]. The postoperative period is a relatively short timeframe compared to the much longer duration of primary tumor development and progression. However, recent mechanistic studies demonstrate that this short period of surgery-induced immunosuppression is critically important in shaping the probability of postoperative metastatic disease [11, 14, 18]. This review will focus on the innate and adaptive immunological pathways that are disrupted by oncologic surgical stress and provide suggestions for rationally combining cancer surgery with immunotherapies to improve immune and treatment outcomes.

## Main

### Molecular and cellular mediators of postoperative immune suppression

**Fig. 1:**
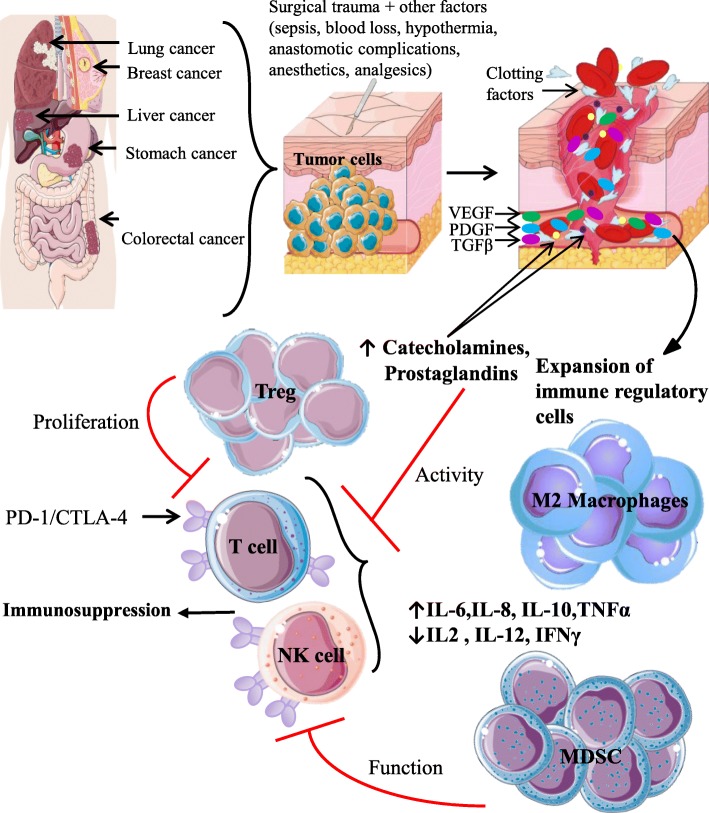
Mechanisms of postoperative immunosuppression. Surgical debulking initiates inflammatory, neuroendocrine and metabolic events, which result in altered cytokine levels (decrease in IL-2, IL-12 and IFN-γ; increase in IL-6/8, IL-10 and TNF-α) and release of growth factors (VEGF - green oval, PDGF - blue oval, TGF-β - pink oval), clotting factors, and stress hormones (catecholamines - yellow circle, prostaglandins - purple circle). While essential for wound healing and pain management, these events lead to the expansion of Tregs, MDSC, and M2 macrophages. Increase in these regulatory immune cells leads to augmented expression of PD-1/CTLA-4, decreased T-cell proliferation, and impaired NK-cell cytotoxicity, resulting in an overall state of immunosuppression. In conjunctions with surgical trauma, other postoperative factors, including sepsis, blood loss, hypothermia, anesthetics, analgesics and anastomotic complications contribute to immunosuppression. Abbreviations: VEGF, vascular endothelial growth factor; PDGF, platelet-derived growth factor; TGF-β; Transforming growth factor beta; Tregs, regulatory T cells; MDSC, myeloid derived suppressor cells*;* PD-1, programmed cell death protein 1; CTLA-4, cytotoxic T lymphocyte associated protein 4

While surgical resection provides effective debulking treatment for solid tumors, the end-result is substantial tissue and vasculature trauma. This is due to unavoidable tumor and normal tissue dissection and the potential removal of organs during major tumor resection [19]. At the cellular level, surgery-induced necrotic cell death leads to the release of sequestered cellular factors. These factors make up the “alarmins” that alert the immune system to the presence of tissue damage. Following the detection of alarmins by pathogen recognition receptors, innate immune cells initiate inflammatory pathways, chemotaxis, antimicrobial defenses, and adaptive immune cell responses [19]. After the early trauma response to tissue injury, pro- and anti-inflammatory responses are temporally regulated by soluble mediators and innate and adaptive immune cells. Cellular immune suppression following cancer surgery has been shown to peak at 3 days and occasionally lasting several weeks [11, 16, 17]. This suppression is multi-factorial, and is characterized by the release of growth factors (VEGF, PDGF, TGF-β), clotting factors, stress hormones (glucocorticoids, catecholamines [20], prostaglandins [21]) and cytokines into the extracellular compartments. Commonly, Th1 cytokines are suppressed following surgery (decrease in IL-2, IL-12 and IFN-γ) [21], leading to a shift towards Th2 immunity (increase in IL-6/8 [20, 21], IL-10 [21] and TNF-α [11]) (Fig. 1). However, several studies have observed an opposite effect of surgery when comparing in vivo plasma to in vitro induced production levels of Th1 cytokines. Using high sensitivity ELISA kits, Ben-Eliyahu’s group observed a significant increase in plasma IFN-γ levels following surgery. It is hypothesized that this inverse pattern of cytokine secretion detected following surgery could be due to differences in the sampling technique. Plasma cytokine measurements reflect physiological amounts of cytokines secreted by the natural composition of cells in vivo, while in vitro-induced cytokine readings are measured from isolated cell populations following non-physiological levels of LPS/PHA stimulation [15]. The overall effect of these secreted factors is the rapid expansion of regulatory myeloid (myeloid derived suppressor cells - MDSC, M2 macrophages) and T regulatory cells (Treg) (Fig. 1). Tissue trauma, in general, triggers a number of changes in phenotype and function, including enhanced activation of Tregs and expansion of MDSC. Following cancer surgery specifically, *Zhou* et al. detected elevated peripheral Treg levels on postoperative day 7 in breast cancer patients undergoing radical mastectomy [22]. In cervical cancer patients undergoing laparoscopy, elevated levels of both MDSC and Tregs lead to a Th1, Th2, Th17 and Treg cytokine imbalance. In these patients, perioperative multi-dose treatment with the COX-2 inhibitor Parecoxib was found to reduce postoperative immunosuppression through restoration of cytokine levels [23]. In contrast to the above studies describing expansion of Tregs, peripheral Treg populations obtained from ovarian cancer patients were observed to diminish significantly at postoperative day 3, followed by an augmentation at day 7. Furthermore, accumulation of Treg populations postoperatively was found to be tumor stage dependent, as patients with early stage I/II tumors showed decrease Treg population, while those with late stage III/IV tumors exhibited greater amounts by comparison [24].

As integral members of the innate immune system, Natural Killer (NK)-cells are involved in the direct killing of cells displaying abnormalities linked to infection, malignancy or transplantation [25, 26]. Immunosurveillance of the host by NK-cells for malignant cells results in direct cytotoxicity and the production of cytokines to enhance the immune response [26]. Postoperative NK-cell cytotoxic dysfunction has been demonstrated in preclinical [11, 27–30] and clinical studies [11, 17, 29]. NK-cell functional impairment is associated with progressive metastatic disease in animal experimental models [4, 11, 31, 32]. In human patients with solid malignancies, inferior NK-cell function following surgery correlates with poor prognosis [33–35]. Even with the numerous reports documenting postoperative NK-cell suppression, very few studies have characterized the underlying mechanism of this impairment [4, 32, 36]. We provided the first in vivo evidence linking surgery to the metastasis of cancers via NK-cells through adoptive transfer of surgically stressed and control NK-cells into NK-deficient recipient mice, showing that surgically stressed NK-cells cannot protect from a lung tumor challenge. The impairment in NK-cell function was also linked to MDSC accumulation [11]. Specifically, postoperative expansion of granulocytic MDSC impair NK-cells through the ROS/arginase I/IL-4Rα axis [37]. In human studies, postoperative NK-cell cytotoxicity was markedly reduced following major surgical resection of the primary tumor in patients with colorectal cancer [11]. The impairment in NK-cell function also directly correlates with MDSC expansion [37] (Fig. 1).

T-cell dysfunction following physical injury and/or surgical trauma has been shown to impair host defenses and increase susceptibility to infection [38–40]. Postoperative dysfunctional T-cell responses have been shown to include the inability to recall antigens, diminished membrane expression of the T-cell receptor (TCR) and loss of the zeta (ζ) chain, decreased proliferation and production of IFN-γ along with other impairments [41, 42]. An important subset of T-cells, the CD8^+^ T-cells has recently been in the spotlight in the cancer immunology/immunotherapy field. We demonstrated the impact of surgical stress on the development and maintenance of an acquired T-cell mediated anti-tumor immune response in the context of adjuvant vaccination. We demonstrated that surgical stress results in reduced proliferation and function as shown by a decrease in the number of CD8^+^ T-cell that produce cytokines (IFN-γ, TNF-α, Granzyme B), in response to dopachrome tautomerase, a tumor associated antigen (TAA). In a prophylactic cancer vaccination model, surgical stress completely abolishes tumor protection conferred by vaccination in the immediate postoperative period. In a clinically relevant surgical resection model, vaccinated mice receiving tumor debulking with a positive margin and additional surgical stress had diminished survival compared to mice with positive margin resection alone. Significantly, MDSC population numbers and functional impairment of TAA-specific CD8^+^ T-cells were altered in surgically stressed mice [12]. Similarly, a mechanistic role for MDSC-induced arginine depletion after physical injury as a cause of global T-cell dysfunction has been described [38]. Translational studies involving cancer patients displayed global reduction in function and number of T cells postoperatively [43]. In addition to these findings, Treg expansion following surgery has been shown to increase the expression of the checkpoint inhibitor PD-1 on T-cells and NK-cells. This is turn promoted up-regulation of caspase-3 and facilitating immunosuppression and apoptosis induced reduction of cytotoxic immune populations [44] (Fig. 1).

### Current combination studies of surgery and immunotherapy

**Fig. 2:**
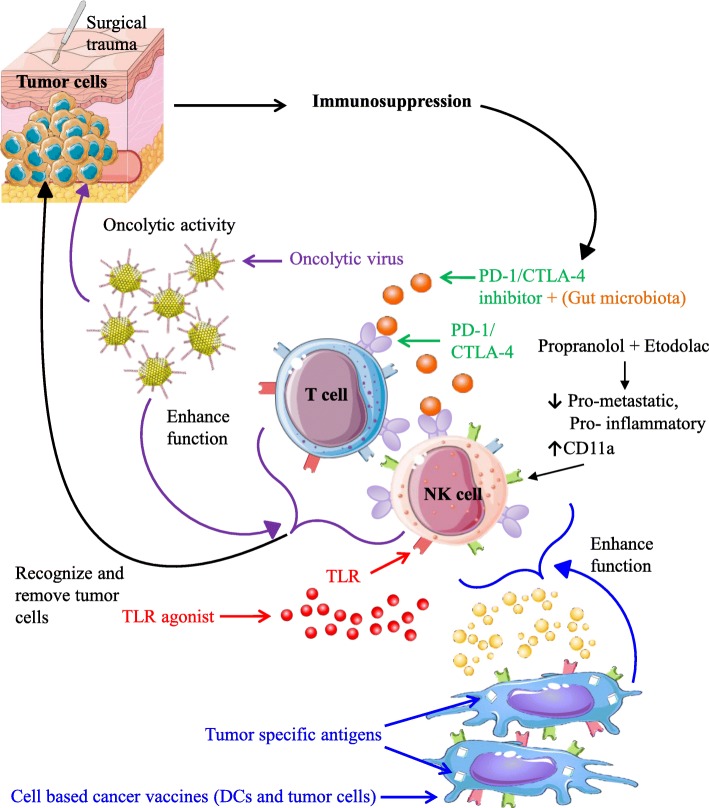
Combination strategies of surgery and immunotherapy. The perioperative time-frame provides a therapeutic window, which can be exploited to reduce postoperative immunosuppression and tumor growth. Perioperative use of Propranolol (β-Adrenergic inhibitor) in combination with Etodolac (COX-2 inhibitor) has been shown to reduce pro-metastatic and pro-inflammatory pathways while enhancing expression of NK-cell activation marker CD11a. Perioperative use of oncolytic viruses demonstrates lytic capability towards tumor cells, while restoring and enhancing NK- and T-cell immune cell function postoperatively. Use of PD-1/CTLA-4 inhibitors (with or without combination with microbiota) have also shown promising effects on postoperative T-cell dysfunction. Similar postoperative beneficial immune effects were observed following DC and tumor cell-based vaccines and TLR agonists. Abbreviations: PD-1, programmed cell death protein 1; DC, dendritic cells; COX-2, Prostaglandin-endoperoxide synthase 2; CTLA-4, cytotoxic T lymphocyte associated protein 4; TLR, toll like receptor

In light of these findings on cancer surgery-induced immune dysfunction, perioperative immune modulation has been attempted to reverse postoperative metastatic disease (Fig. 2). Emerging preclinical and clinical studies reveal that postoperative immune suppression is reversible. The perioperative period (the time before and after surgery) has been described as a window of opportunity for cancer cells to proliferate and metastasize [16, 45]. Patients recovering from surgery during this critical period have traditionally not received adjuvant chemotherapy or radiotherapy due to the detrimental effect of these interventions on wound repair and further immune suppression. On the other hand, the perioperative period potentially provides a window of opportunity to strengthen the immune system and attenuate the development of cancer recurrences [16] . We will discuss in this review promising and rational combination of surgery and immunotherapy that could reduce or prevent recurrent tumors following cancer surgery.

### Cytokine therapy and TLR agonists

Early immunotherapies such as the recombinant cytokines IL-2, IL-12, or IFN-α have been used to stimulate expansion and activation of effector lymphocytes [46]. Although effective in reducing immune suppression and metastatic disease in animal models and in early phase clinical trials, severe and systemic toxicity, pyrogenic reactions and high dose related septic shock reactions have been observed. Thus, the delivery of these cytokines have faced considerable obstacles for therapeutic use in the perioperative setting [47–49]. Overcoming these deleterious effects, modified synthetic agents expressing reduced or limited toxicity with highly effective, multi-cytokine responses have recently been approved for clinical use [47, 50]. Activating NK-cells, B-cells and plasmacytoid dendritic cells [50], the toll-like receptor-9 (TLR-9) agonist CpG oligodeoxynucleotide demonstrated efficacy in preclinical models in both prophylactic and therapeutic treatment settings [50, 51]. Significantly decreasing metastatic expansion in rats, the synthetic TLR-4 agonist glucopyranosyl lipid-A (GLA-SE) functions through a primarily NK-cell-mediated mechanism increasing both NK-cell number and function [47]. Designed to enhance Th1 immunity with limited adverse effects such as those observed with other biological TLR-4 ligands [48, 49], GLA-SE treatment lead to increased plasma levels of IL-15, IFN-γ, and plasma levels of IL-6 but not IL-1β, while not affecting physical or behaviour changes in rats [47].

Despite the paucity of data and clinical limitations, several clinical trials using preoperative low dose recombinant IFN-α [52] and IL-2 [53–56] have demonstrated less NK- and T-cell suppression and improved prognosis following surgery in patients undergoing colorectal cancer [56] and hepatic metastasis resection [57]. In a study using preoperative IL-2, 86 colorectal cancer (CRC) patients with stage II/III disease were randomized to receive low dose IL-2 twice a day for 3 consecutive days before surgery or no treatment. There were significantly fewer recurrences in the IL-2 group (21.4% vs 43.1%, *p* < 0.05) and improved overall survival (OS) at a median follow-up of 54 months [56]. In another IL2 perioperative study, 50 CRC surgical patients with Stage IV disease were randomized to receive preoperative low dose IL-2 or no treatment. The median progression-free survival (PFS) and OS were significantly longer in the preoperative IL-2 treated group [57]. While these studies were not powered to assess oncologic outcomes, a Phase II trial in 120 surgical patients with renal cell carcinoma demonstrated a significant improvement in 5 year PFS with preoperative IL-2 treatment (74% vs. 62%, *p* = 0.02) [53]. Importantly, in all of these studies, preoperative IL-2 was well tolerated with adverse events limited to pyrexia (Grade I-III). These preclinical and clinical results are promising and suggest that modified or low dose recombinant cytokine/TLR agonist therapy that can enhance the immune system warrant further study for perioperative administration (Fig. 2).

### β-adrenergic blockers and COX2 inhibitors

In a recently completed proof-of-concept trial, 38 early-stage breast cancer patients received perioperative Propranolol (β-adrenergic inhibitor) and Etodolac (COX-2 inhibitor) combination treatment to inhibit the release of surgery-induced catecholamines and prostaglandins. Transcriptome profiling of patient tumors revealed reduced pro-metastatic and pro-inflammatory pathways, providing the rationale to pursue future large clinical trials to evaluate the clinical impact of perioperative Propranolol and Etodolac. These beneficial effects were suggested to occur through an NK-cell mediated mechanism, as circulating NK-cells expressed enhanced expression of tumor cell lysis promoting marker CD11a [58] (Fig. 2). In colorectal hepatic metastatic mouse models, both mild (small incision) and extensive (small incision and laparotomy) surgical procedures displayed proportional increase in metastatic susceptibility, which subsided significantly following combination Propranolol and Etodolac treatment [59]. While the beneficial effects of these inhibitors are promising, future safety and efficacy trials are required in order to understand the effects of perioperative Propranolol and Etodolac on patients with pre-existing contraindications and co-morbidities, including diabetes, asthma, cardiovascular and autoimmune disease in order to modulate drug dose, duration and concentration [58]*.*

### Checkpoint inhibitors

Checkpoint inhibitors against PD-1 have been shown to relieve postoperative T-cell dysfunction. However, while such inhibitors increased IFN-γ production, T-cell proliferation remained limited. To improve upon this, the use of prostaglandin inhibitors in combination with PD-1 inhibitors was found to restore postoperative T-cell function completely [60]. Utilizing the functional properties of platelets, in situ activation of platelets following adhesion combined with anti-PD-1 was found to reduce residual tumor cell presence and formation of metastatic loci in both primary melanoma and triple negative breast cancer (TNBC) patients through robust activation of T-cell mediated antitumor immunity [61]. Similar to the effects of anti-PD-1 treatment in the postoperative period, increased T-cell activation has been demonstrated following the administration of CTLA-4 inhibitors in preclinical and clinical metastatic settings. Padmanee Sharma’s group demonstrated enhanced expression of the inducible costimulatory molecule (ICOS) on CD4^+^ T-cells in both peripheral and tumor tissue populations in the setting of neoadjuvant delivery of ipilimumab in urothelial carcinoma. In addition, an increase in tumor infiltration of CD3^+^, CD8^+^, and CD4^+^ T-cells expressing granzyme was reported. Following a retrospective analyses in a separate patient group with either unresectable stage III or metastatic/recurrent stage IV melanoma, improved overall survival correlated with a consistent increase in CD4^+^ICOS^hi^ T- cell populations at 12 weeks following 4 dosing cycles of ipilimumab [62].

In a CT26 lung metastasis mouse model, the combination treatment of ipilimumab with poxvirus MVA-BN-HER2 increased overall survival to greater than 100 days. This significant increase in survival time was associated with the quality of the immune response, as the presence of the virus was observed to induce the expression of IFN-γ, TNF-α, and IL-2 on CD8^+^ T-cells [63]. Despite the promising results for delivering checkpoint inhibitors to recover surgery-induced immune dysfunction, the expression of PD-1 has been shown to vary significantly on T-cells and NK-cells between different postoperative days, which could impact the efficacy of checkpoint blockade in the postoperative period.

Overcoming these limitations, neoadjuvant treatment with anti-PD-1 and anti-CD137 was shown to significantly enhance overall survival efficacy beyond 100 days in both murine 4 T1.2 TNBC and E0771 mammary carcinoma models compared to adjuvant treatment. This significant survival enhancement was associated with IFN-γ production and increased presence of gp70 tumor-specific CD8^+^ T-cells in the blood following treatment and well beyond surgery [64]. Similarly, a small study evaluating the safety and efficacy of neoadjuvant anti-CTLA-4 inhibitor ipilimumab in regionally advanced melanoma patients demonstrated the immunomodulatory role of the inhibitor on MDSC, Treg and effector T-cell populations in both the circulation and tumor microenvironment. Six weeks post-treatment, a significant decrease in circulating MDSC populations was associated with improved progression free survival (PFS). Unexpectedly, increased circulating Treg populations, but not tumor-associated populations, improved PFS. Further improvement was associated with an increase in tumor infiltrating and activated CD4^+^ and CD8^+^ T-cells populations, and generation of memory T-cells [65]. In a pilot study examining the effect of neoadjuvant anti-PD-1 inhibitor nivolumab in resectable non-small cell lung cancer patients, major pathological responses were observed in both PD-L1 positive and negative tumors that were associated with increased proliferation of both tumor infiltrating and peripheral T-cells. In addition, rapid expansion of mutation-associated, neoantigen-specific T-cells was observed as early as 2 to 4 weeks following initial nivolumab administration demonstrating the additional benefit of neoadjuvant treatment [66].

Altogether, these collective preclinical and translational studies on perioperative administration of checkpoint inhibitors demonstrate significant enhancements in antitumor responses. We speculate that neoadjuvant/preoperative injection of anti-PD-1 inhibitors might be advantageous to activate tumor infiltrating T-cells prior to surgery and to avoid the reduction of PD-1 expression on immune cells in the postoperative period. However, further testing of checkpoint inhibitors in combination with surgery in various tumor types and larger cohorts of patients will be required to asses the relative contribution of various immune cell subsets to improved patient prognosis.

### Oncolytic viruses

Compared to cytokines and TLR agonists, oncolytic viruses (OV) like regular viruses provoke a more physiological and multi-dimensional immune response following their in vivo delivery [11, 67]. We and others have shown that OV can engage and mature conventional dendritic cells (DC) amongst other innate cells, which in turn activate NK- and T-cells [11, 67–69]. The complex constellation of cytokines and chemokines released in response to a virus infection would be very difficult to characterize and reproduce as a cytokine cocktail for perioperative injection. Additionally, the OV provides the benefit of direct cytolysis of metastatic tumor cells on top of its immune stimulating abilities [67] (Fig. 2). Lastly, the enhanced release of growth factors such as vascular endothelial factor (VEGF) following surgery, may allow for better infection and replication of OV in tumor cells [70, 71]. Therefore, there is a compelling rationale to attempt OV therapy in the perioperative period.

Given that OV can stimulate NK-cells and cancer surgery impairs NK-cells, we investigated the capacity of preoperative OV to prevent the development of postoperative metastases secondary to postoperative NK-cell dysfunction. In preclinical mouse models of solid tumors with major surgical resection, we determined that preoperative administration of oncolytic vaccinia virus, parapox ovis (ORF) and rhabdoviruses (Maraba MG1, VSVd51) can recover postoperative NK-cell dysfunction followed by reduction in postoperative metastases [11, 67]. We determined that the reduction in tumors was indeed due to NK-cell mediated tumor lysis following its activation by OVs [11]. Mechanistically, we demonstrated that NK-cell activation in the context of OV infection is preceded by conventional (DC) activation and MDSC expansion [11, 67] .

In human studies, a single intravenous (iv) dose of oncolytic vaccinia virus before surgical resection resulted in improved postoperative NK-cell cytotoxicity in patients with metastatic colorectal tumors to the liver [11]. Although this study was not powered to assess prognosis, these results demonstrated for the first time that oncolytic vaccina virus markedly increases NK-cell activity in cancer surgery patients. In the same patient population and clinical setting, iv delivery of oncolytic reovirus resulted in the identification of reovirus genome in resected liver tumor tissue, but not normal liver tissue. Significantly, surgical patients suffered most commonly from mild flu-like symptoms with no reported grade 3 or 4 toxicities [72]. In a separate study of oncolytic Herpes Simplex Virus (HSV) treatment, virus was injected intratumorally before and after surgery in patients with recurrent Glioblastoma Multiforme. Similar to the reovirus study, viral replication and immune cell infiltration was detected in resected tumors. Importantly, patients tolerated HSV well and did not suffer from virus related encephalitis [73]. While the use of perioperative OV in clinical studies has shown promising effects on reversing surgery induced immunosuppression through lytic activity and inducing immune response, there are theoretical safety concerns associated with viremia in human cancer surgical patients. For example, reversion of attenuated OV back to wild type virus may increase non-specific targeting of healthy cells. Furthermore, concerns associated with potential viral spread to the operating team may limit the use of OV in combination with cancer surgery [67]. However, the human reports outlined above using a variety of OV in numerous solid tumors with minimal side effects demonstrate the feasibility and safety of perioperative OV administration into cancer surgery patients.

### Cancer vaccines

Cancer vaccines based on modified DC have also been administered in combination with surgery. Stimulation of DCs through recombinant human granulocyte-macrophage colony-stimulating factor (GM-CSF), IL-4 and TNF-α followed by sensitization with autologous tumor cells was found to significantly increase postoperative CD8^+^ T-cell production, in addition to IL-2 and IFN-γ secretion. The overall effect was the induction of anti-tumor responses towards various tumor antigens and reduction of tumor proliferation [74]. To improve tumor targeting and patient survival, sequential postoperative combination of DC vaccines with cytokine-induced killer cell therapy (CIK) was used. This augmented the secretion of Th1 cytokines with a significant increase in IL-12 and IFN-γ in both gastric and colorectal cancer patients [75]. In similar studies using just postoperative delivery of autologous CIK cells, *Pan* et al.*,* displayed improved overall survival and disease-free survival in TNBC patients. Mechanistically, the CIK based vaccine resulted in the intratumoral release IL-2, IFN-γ, and TNF-α thereby increasing immunosurveillance and antitumor immunity [76] (Fig. 2).

Using oncolytic Newcastle Disease Virus (NDV) to infect autologous tumor cells ex vivo from glioblastoma, colorectal and renal cell carcinoma patients, followed by postoperative injection of this OV modified tumor vaccine, researchers found enhanced survival in vaccinated patients compared to unvaccinated cohorts [43, 77, 78]. By means of an oncolytic rhabdovirus engineered to express TAA and using a prime-boost tumor vaccination approach, it was determined that TAA specific T-cell immune responses can be generated to protect mice from melanoma tumor challenge and lead to a significant diminution in lung metastases. Specific in vivo depletion of cytotoxic CD8^+^ T-cells during the boost vaccination abolished the therapeutic efficacy of the vaccine, highlighting their mediating role [79–81].

The perioperative use of DC and OV-based vaccines also presents a set of delivery challenges. As DC sensitization with autologous tumor cells would require cells from the tumor itself, a proper representation of the unique and specific tumor antigens in the tumor can only be achieved through surgical debulking. Using the resected tumor bulk to stimulate DCs would provide greater specificity and efficacy of metastatic tumor antigen targeting [74]. Therefore, a postoperative adjuvant delivery strategy of DC-based tumor vaccines makes the most sense. OV-based tumor vaccines present the same set of challenges as OV therapy. There exists the potential for a postoperative systemic inflammatory response, the risk of viral spread to members of the operating room team and risk of meningitis with epidural analgesia if the OV based vaccine is administered prior to surgery. However, the NDV based vaccine approach has not resulted in any adverse events for treated patients and the prime boost approach with oncolytic rhabdovirus in human late stage melanoma patients is ongoing with no safety concerns reported [82].

### Other perioperative factors that contribute to immune suppression

The use of anesthetics and analgesics are necessary components of surgical resection for pain management. However, these agents have been shown to reduce NK- and T-cell proliferation and function in both rat models and healthy human volunteers via the release of endogenous opioids and stress related molecules [83]. Using clinically relevant doses of morphine, both direct and antibody dependent cellular cytotoxicity-mediated NK-cell killing was not only shown to decrease in healthy human volunteers [84], but to also accelerate human breast tumor growth in in vivo xenogeneic mouse models through promotion of cell cycle progression, angiogenesis and endothelial cell proliferation [85]. Chemical derivatives of morphine, such as fentanyl have been shown to increase the development of rat lung metastasis due to reduced NK-cell cytotoxicity [86]. In contrast to these findings, morphine has also been reported to inhibit metastatic spread and induce NK-cell activation under postoperative conditions using rat tumor models [87]. Similarly, intravenous administration of fentanyl in healthy human volunteers was found to significantly enhance NK-cell cytotoxicity, in addition to increasing CD16^+^ and CD8^+^ lymphocyte numbers [88] (Fig. 1).

Alongside perioperative pain management, further perioperative factors such as intraoperative blood loss, hypothermia and postoperative sepsis have been shown to contribute to postoperative immune suppression. Modern surgical practice ensures minimization of these adverse outcomes, however, despite precautions, 6–10% of advanced cancer patients experience blood loss [89], 8.5% of cancer related deaths are correlated to development of sever sepsis [90], and 70% of cancer surgical patients experience hypothermia (defined as core body temperature < 36^°^*C*) [91]. The occurrence of these complications has been outlined in multiple clinical studies to reduce cancer specific survival following surgery. The occurrence of hypothermia was associated with increased risk of early complications, infection, and reduced overall survival in stage IIIC and IV ovarian cancer patients undergoing abdominal surgery [92]. In colon cancer bearing rat models, perioperative hypothermia was found to accelerate tumor growth [93], in addition to suppressing NK-cell activity [27]. In contrast, we recently demonstrated that neither intraoperative blood loss or hypothermia affect the prometastatic effects of surgical stress [94]. However, the development of postsurgical sepsis enhances postoperative tumor progression through an NK-cell-mediated mechanism, which was relieved following the addition of poly(I:C), a double-stranded RNA mimetic [94].

Specific to CRC surgery, additional perioperative factors such as changes in the gut microbiome of the patient leading to depletion of short-chain fatty acids [95] and the development of anastomotic complications have been associated with increased risk of local tumor recurrence [96] (Fig. 1). In vitro treatment of MDA-MB-231 cancer cells with peritoneal fluid from CRC patients experiencing anastomotic complications was shown to impact both tumor invasiveness and proliferation [97]. These oncological stimulatory effects were suggested to result from the proinflammatory response towards peritoneal infection, facilitating tumor recurrence through secretion of multiple tumor stimulatory factors including IL-6 and VEGF [98]. In mouse models, gut microbiota was found to play a significant role in modulating the immune response towards checkpoint inhibitor immunotherapy. Following oral administration of *Bifidobacterium* to B16. SIY melanoma mice, Sivan et al.*,* demonstrated equal tumor control compared to anti-PD-1 treatment and significantly enhanced antitumor response in combination with anti-PD-1. Enhanced DC function leading to increased CD8^+^ T-cell priming and tumor infiltration was suggested to be the underlying mechanism of combination therapy [99]. Similarly, modulation of CTLA-4 efficacy in MCA205 sarcoma mouse models and patients with metastatic melanoma and non-small cell lung carcinoma were found to be dependent on the presence of *B. fragilis* or *B. thetaiotaomicron* influencing antitumor response through IL-2 dependent Th1 immunity, while simultaneously limiting anti-CTLA-4-mediated intestinal adverse effects [100] (Fig. 2).

## Conclusions

### Perioperative window of opportunity for immunotherapy

Cancer surgery is the standard-of-care for patients with solid tumors. Despite its curative intent, the majority of patients relapse with postoperative disease. Because the patient seems to be at maximum risk for immunosuppression during the immediate postoperative period, this may represent a therapeutic window of opportunity during which novel immunomodulatory treatments aimed at reducing perioperative tumor growth may be used. There are currently no standard perioperative anti-cancer therapies aimed at preventing postoperative metastases due to concerns associated with wound repair and patient recovery. Emerging mechanistic data in both preclinical and translational studies using novel therapies that can activate both the innate and adaptive immune responses have shown promise. Early clinical trials confirm the feasibility of these strategies, but these therapies must be rigorously tested for safety and efficacy and then translated into rationally designed clinical trials powered to assess oncologic outcomes. Through further mechanistic investigation on sequential combination of immunotherapy with surgery and creation of precise treatment profiles associated with individual patient responses, we envision a future where the protection of cancer patients against postoperative tumor growth becomes part of the accepted therapeutic paradigm. Based on the described studies, we propose a practice-changing paradigm – that cancer patients bearing solid tumors may be further protected against recurrent disease by receiving perioperative immunotherapy in combination with standard-of-care surgery. This combination treatment strategy has the potential to improve survival in countless cancer surgical patients each year.

## Abbreviations

CIK: Cytokine-induced killer
COX-2: Cyclooxygenase 1
CpG: Cytosine phosphate Guanine oligodeoxynucleotides
CRC: Colorectal cancer
CTLA-4: Cytotoxic T lymphocyte associated protein 4
DC: Dendritic cell
GLA-SE: Glucopyranosyl lipid adjuvant squalene emulsion
GM-CSF: Granulocyte macrophage colony stimulating factor
HSV: Herpes Simplex Virus
ICOS: Inducible T cell costimulator
IFN-α: Interferon alpha
IFN-γ: Interferon gamma
LPS/PHA: Lipopolysaccharide/ Phytohaemagglutinin
MDSC: Myeloid derived suppressor cell
NDV: Newcastle Disease Virus
NK-cell: Natural Killer cells
ORF: Parapox ovis
OS: Overall survival
OV: Oncolytic viruses
PD-1: Programmed cell death protein 1
PDGF: Platelet derived growth factor
PFS: Progression free survival
PFS: Progression-free survival
TAA: Tumor associated antigen
TCR: T-cell receptor
TGF-β: Transforming growth factor beta
TLR: Toll like receptor
TNBC: Triple negative breast cancer
TNF-α: Tumor necrosis factor alpha
Tregs: T regulatory cells
VEGF: Vascular endothelial growth factor
